# PtoHsfB1 regulates growth and salt response by affecting ABA biosynthesis in *Populus tomentosa*

**DOI:** 10.48130/forres-0026-0005

**Published:** 2026-02-28

**Authors:** Kunjin Han, Huayu Si, Ye Li, Jifeng Yan, Yousry A. El-Kassaby, Yizhe Cheng, Houyin Deng, Jie Liu, Yuhan Sun, Yun Li, Ye Zhao

**Affiliations:** 1 State Key Laboratory of Tree Genetics and Breeding, National Engineering Research Center of Tree Breeding and Ecological Restoration, Engineering Technology Research Center of Black Locust of National Forestry and Grassland Administration, College of Biological Sciences and Technology, Beijing Forestry University, Beijing 100083, China; 2 Cangzhou Natural Resources and Planning Bureau, Cangzhou 061001, China; 3 Department of Forest and Conservation Sciences Faculty of Forestry, The University of British Columbia, 2424 Main Mall, Vancouver, BC V6T 1Z4, Canada

**Keywords:** *PtoHsfB1*, Repressor, Growth, ABA synthesis, Salt stress

## Abstract

Poplar is an important commercial timber species and a model organism for forest molecular biology. Here, we identified PtoHsfB1, a subgroup B heat shock transcription factor from *Populus tomentosa* that is homologous to AtHsfB1 and PtrHsfB1, and contains the conserved repression motif '-LFGV-'. To elucidate its function, transgenic poplars overexpressing *PtoHsfB1* were generated. Overexpression significantly enhanced growth rate and biomass accumulation. Histological analyses revealed increased cambial cell layers and enlarged phloem caps, indicating a positive role in cambial activity and phloem development. In roots, *PtoHsfB1* promoted growth by suppressing abscisic acid (ABA) biosynthesis. Conversely, *PtoHsfB1* overexpression reduced salt stress tolerance, as evidenced by increased oxidative damage under salt stress conditions. Collectively, these results show that *PtoHsfB1* plays a dual regulatory role by promoting growth but negatively regulating salt stress responses, highlighting its potential application in improving poplar phloem yield and soil conservation.

## Introduction

Poplar trees possess high-quality fibers and rapid growth rates, making them a valuable renewable resource for paper and pulp production, industrial cordage, and soil erosion prevention^[1,2]^. The availability of complete genome databases and stable genetic transformation systems has established poplar trees as a model organism for studying woody plants^[3,4]^. *Populus tomentosa* (*P. tomentosa*), a tree native to northern China, is characterized by fast growth, a straight trunk, broad environmental adaptability, and longevity^[3,5]^. The application of modern biotechnology to enhance secondary growth and wood quality in *P. tomentosa* offers a practical approach to boosting biomass yield and timber quality^[6]^. In the annual herbaceous model plant *Arabidopsis thaliana* (*A. thaliana*), the heat shock factor (Hsf) family is extensively involved in plant growth and development as well as responses to various stresses. For example, *Hsfa* mutants exhibit defects in seed development^[7]^, while overexpression of *HsfA* enhances combined resistance to heat stress, hypoxia, and salt stress^[7]^. Additionally, studies have shown that HsfB plays a crucial role in recovery following heat stress^[8]^. Although substantial progress has been made in *A. thaliana*, research on Hsf function in woody plants remains relatively scarce. In particular, the role of HsfB family members in tree growth and stress responses is still poorly understood. Therefore, expanding and deepening research on HsfB members in woody plants is essential for a comprehensive understanding of Hsf family functions.

The Hsf family constitutes a group of transcription factors present in all eukaryotic species, including fungi, plants, and mammals^[9]^. Plant Hsfs are classified into three groups, A, B, and C^[10,11]^. Class HsfA members are characterized by transactivation activity and play a crucial positive regulatory role in heat stress response^[8]^. In contrast, class B and C members generally lack intrinsic transcriptional activation activity. Most class B Hsfs (with the exception of HsfB5) contain a conserved -LFGV- motif that functions as a transcriptional repressor^[12,13]^. Members of class A Hsfs play a central role in regulating stress resistance. For instance, *HsfA1* enhances heat tolerance in both *A. thaliana* and tomato^[14,15]^. Class B members also play important roles in stress responses. HsfB1 has been shown to positively modulate stress tolerance despite functioning as a transcriptional repressor^[16]^. In *A. thaliana*, HsfB1/B2b interacts with class A Hsfs to regulate the termination of heat shock response^[17]^. In maize, natural variations in the *ZmHsf21* promoter (HsfB family) improve cold tolerance through the regulation of lipid metabolism^[18]^. Despite the well-documented involvement of several HsfB members in stress resistance, their roles in plant growth and development remain largely unexplored^[19]^. To date, only *Arabidopsis AtHsf4* and poplar *PagHsf4* are known to function in inhibiting plant growth^[20,21]^.

The plant hormone abscisic acid (ABA) is a sesquiterpene compound containing 15 carbon atoms^[22]^. Plants synthesize ABA primarily through an 'indirect pathway' that depends on carotenoid metabolism^[22]^, with key regulatory enzymes including NCED, ZEP1, ABA2, and AAO^[22]^. ABA is generally considered a growth inhibitor, as it suppresses growth, reduces root elongation, and decreases fresh weight^[23]^. Research has demonstrated that rapid subcellular relocalization of SnRK1 is necessary for the ABA-induced suppression of cell proliferation in the *A. thaliana* root meristem^[24]^. In rice, ABA inhibits primary root elongation yet promotes root thickening^[25]^. Additionally, ABA accumulation under abiotic stress conditions plays a critical role in reshaping water relations and regulating growth^[26]^. ABA is essential for plant defense against salt stress and is among the most important stress-responsive hormones^[27,28]^. Under salt stress, ABA signaling buys time for root adaptation by delaying developmental processes^[29]^. NCED3, a salt-sensitive gene, encodes the rate-limiting enzyme in ABA biosynthesis^[30]^. Similarly, by regulating ABA levels and maintaining ROS homeostasis, OsNCED4 promotes rice resistance to salt and cold stress^[31]^.

This study demonstrates that overexpression of *PtoHsfB1* in *P. tomentosa* significantly promotes growth but reduces salt tolerance. Further analyses indicate that this phenotype is likely due to decreased ABA levels mediated by *PtoHsfB1* overexpression. Collectively, these findings suggest that PtoHsfB1 plays a key role in regulating growth and salt response in poplar.

## Materials and methods

### Phylogenetic tree construction and sequence analysis

The sequences of 21 Hsf members from *A. thaliana* were retrieved from the PlantTFDB5.0 database (http://planttfdb.cbi.pku.edu.cn). To perform sequence alignment, the Muscle method was used to align the Hsf members of *P. tomentosa*. A phylogenetic tree was constructed using the Neighbor-Joining (NJ) method with the Poisson model and 1,000 bootstrap repetitions in MEGA 5.05^[32]^. For protein sequence comparison between *P. tomentosa* and *P. trichocarpa*, the alignment was performed using DNAMAN 6.0 (https://www.dnaman.net). The phylogenetic tree was visualized using TBtools^[33]^.

### Transcriptional activation activity detection

Transcriptional activation was assessed using the Yeast Transcription Assay kit (Coolaber, Beijing, China). The coding sequence of *PtoHsfB1* was inserted into the pGBKT7-VP16 yeast expression vector. The VP16 protein, derived from the herpes simplex virus, acts as a transcriptional activation factor to increase the expression of downstream genes^[34]^.

### Plant materials

The plant material used in this study was the 'Yixian' clone line (*P. tomentosa*). Shoot tips ranging from 1.5 to 2 cm in length were excised from *P. tomentosa* plantlets and cultivated in sterile tissue culture flasks with a rooting medium containing 1/2 MS, 0.4 mg/L IBA, 20 g/L sucrose, and 6.5 g/L agar. The cultures were maintained under a 16-h light (4,500 lux), and 8-h darkness cycle at 25 °C^[10]^. After 30 d, the plantlets were transferred to a soil mixture consisting of vermiculite, turfy soil, and potting soil (1:3:1 ratio) in a greenhouse under similar conditions to those in the tissue culture room^[10]^.

### Vector construction and genetic transformation

The amino acid sequences of PtoHsfB1 homologs were obtained from the National Center for Biotechnology Information database. The full coding sequences of *PtoHsfB1* were amplified from the 'Yixian' clone using the primers listed in Supplementary Table S1, and subsequently integrated into the pBI121 vector to create the *35S::PtoHsfB1* fusion construct. For *P. tomentosa* genetic transformation, the fusion vector was transferred to the GV3101 strain of *Agrobacterium tumefaciens*^[35]^.

### Growth indicator analysis

For morphological analysis, one-month-old and three-month-old wild-type (WT), and transgenic plants were assessed. Key growth parameters, including plant height, fresh weight, and dry weight, were recorded. Plant growth was photographically documented at multiple stages.

### Histological analyses

For histological analysis, three-month-old transgenic and WT plants were selected. Stem segments (approximately 0.5 cm long) were embedded in 7% agarose and cut into 50-μm-thick cross-sections using a vibratome microtome (Leica VT1000S, Nussloch, Germany). The sections were then stained with a 0.05% (v/v) solution of toluidine blue O (TBO). The stained sections were observed using an Olympus BX51 digital microscope. Xylem and cambium cell dimensions were measured using ImageJ software^[36]^.

### RNA-seq and quantitative real-time PCR (RT-qPCR) analysis

Total RNA was extracted from root tissue of one-month-old WT, and *PtoHsfB1* OE1 and OE8 plants (Vazyme, Nanjing, China). Three biological replicates were used for each sample. RNA sequencing libraries were constructed from high-quality RNA, and raw sequencing data were filtered^[37]^.

Illumina HiSeq 2000 paired-end sequencing was used. The clean data was mapped to the *Populus* genome (PRJNA613008-SRA-NCBI) using HISAT2, and gene expression levels were quantified using FPKM^[38]^. Differentially expressed genes (DEGs) were identified using DEseq2^[39]^. Gene ontology (GO) enrichment analysis of the DEGs was performed using KOBAS v2.1.1 with a *p*-value cutoff of 0.05^[40]^.

Reverse transcription and RT-qPCR analysis were performed using the RNA isolated from the aforementioned root tissues (Vazyme, Nanjing, China). The internal control utilized to normalize the amount of gene expression was the *P. tomentosa Actin* gene. Supplementary Tables S1 and S2 provide a list of primers used in RT-qPCR.

### Salt treatment

For the salt stress experiment, one-month-old *P. tomentosa* plants were chosen. The control group was treated with a nutrient solution, while the experimental group was treated with a nutrient solution containing 200 mM NaCl. After 10 d, the 3^rd^ and 4^th^ fully expanded leaves from six plants were collected, then immediately immersed in liquid nitrogen and kept at −80 °C. Each treatment group consisted of three biological replicates, with two plants per replicate.

### Protein content determination by BCA assay

Protein content in fresh plants was determined using the BCA Protein Assay Kit (Cowin Biotech Co., Ltd., Jiangsu, China). The plant tissues were homogenized in phosphate buffer before the assay.

### ABA content determination

ABA content in fresh stem and root tissues was quantified using a Plant Abscisic Acid (ABA) test (NJJCbio, Nanjing, China). The protein content, as determined by the BCA assay, was used to normalize the ABA levels.

### DAB and NBT staining

For reactive oxygen species (ROS) detection, the leaves were punched into 1 cm diameter discs following salt treatment for 10 d. The discs were then stained overnight at room temperature with either 3,3' N-diaminobenzidine tetrahydrochloride (DAB) solution (1 mg/m L, pH 3.8), or nitro blue tetrazolium (NBT) (0.5 mg/mL, pH 7.8) to detect H_2_O_2_ and O_2_^−^, respectively^[40]^. Following staining, the discs were destained by boiling in ethanol to remove chlorophyll, cooled to room temperature, and then imaged.

### Determination of H_2_O_2_ and MDA content

To assess oxidative stress, H_2_O_2_ content in fresh leaf tissues was determined using a Hydrogen Peroxide Content Assay Kit (NJJCbio, Nanjing, China). The absorbance of each sample was measured at 405 nm.

Malondialdehyde (MDA) content was determined using a Plant assay kit (NJJCbio, Nanjing, China) following homogenization of the tissues in Reagent V, with absorbance measured at 532 nm.

### Statistical analysis

All experiments were conducted with at least three biological replicates. Results were presented as mean ± SE. Statistical analysis was performed using SPSS 22.0 (IBM, Armonk, NY, USA), with significant differences between groups assessed using Duncan's multiple range test. Significant differences are indicated by * (*p* < 0.05) and ** (*p* < 0.01).

## Results

### Identification of PtoHsfB1 in *P. tomentosa*

**Figure 1:**
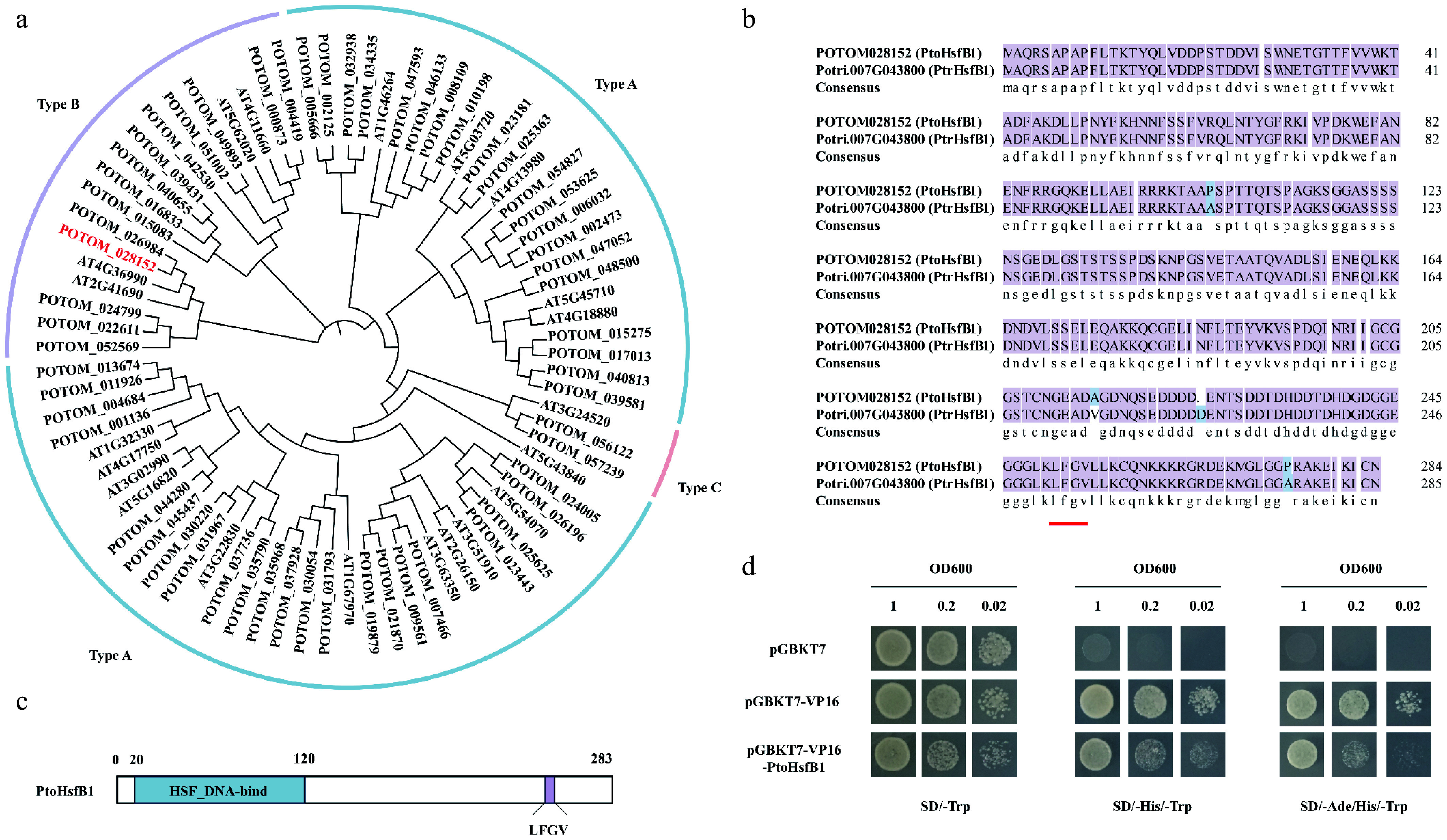
The protein sequence analysis of PtoHsfB1 in *P. tomentosa*. (a) A NJ phylogenetic tree of Hsfs from *A. thaliana* and *P. tomentosa* was generated. The line colors blue, purple, and pink represent subgroups A, B, and C, respectively. (b) Comparison of the protein sequence of PtoHsfB1 and PtrHsfB1. The red underlined part represents the core tetrapeptide of the subgroup B transcriptional repressor Hsf. (c)The protein structure of PtoHsfB1. The blue part represents the Hsf DNA binding domain, and the purple part represents the core tetrapeptide 'LFGV' of the repressor domain. (d) Yeast transcriptional activation assay for PtoHsfB1 (negative control: pGBKT7; positive control: pGBKT7-VP16).

The poplar genome was searched using the hidden Markov model (HMM) of Hsf (PF00447) as a guide, and after multiple confirmations using Pfam and NCBI-CDD, 58 Hsf members were identified. To illustrate the evolutionary links between the species, we constructed a phylogenetic tree for *P. tomentosa* and *A. thaliana* that included 58 PtoHsf and 21 AtHsf proteins (Fig. 1a). Class B member PtoHsfB1 (POTOM_028152) is homologous to *A. thaliana* protein AtHsfB1 (AT4G36990). Additionally, PtoHsfB1 exhibited high homology with *P. trichocarpa* PtrHsfB1 (Potri.007G043800), with a sequence similarity as high as 98.60% (Fig. 1b). The protein sequence of PtoHsfB1 contained 283 amino acid residues, with its Hsf DNA binding domain located between the 20^th^ and 120^th^ amino acid residues. The core tetrapeptide 'LFGV' is located at the C-terminus (Fig. 1c). Subsequently, PtoHsfB1 has transcriptional repressive activity, according to transcriptional activation tests conducted in yeast (Fig. 1d), indicating that the core tetrapeptide of C-terminus is essential for the transcriptional inhibitory function of PtoHsfB1.

### Genetic transformation and transgenic identification of *PtoHsfB1*

**Figure 2:**
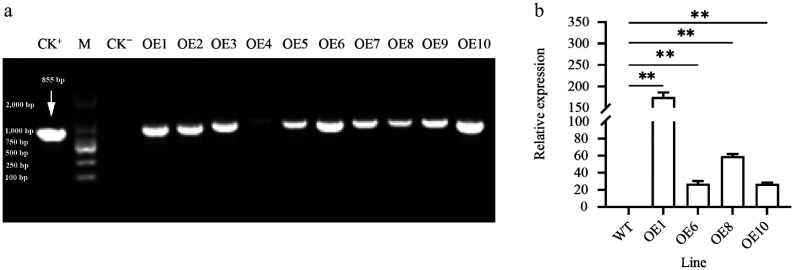
Expression of *PtoHsfB1* in leaves of plantlets. (a) PCR amplification of *PtoHsfB1* from WT and different transgenic lines. CK^+^: Plasmid pBI121-*PtoHsfB1*, M: DL2000 DNA marker, CK^−^: untransformed line. (b) Relative quantification of RNA in transgenic plants and WT plants.

To explore HsfB1 function, transgenic poplars (*35S::PtoHsfB1*) carrying the *PtoHsfB1* gene controlled by the cauliflower mosaic virus 35S promoter were created through *Agrobacterium*-mediated transformation. After screening with kanamycin, a total of 10 transgenic lines were verified through DNA identification. We randomly selected four transgenic lines (OE1, OE6, OE8, and OE10) for RT-qPCR. The results suggested that the overexpression (OE) lines OE1 and OE8 had the highest expression levels of *PtoHsfB1* (Fig. 2). Therefore, these two transgenic lines (OE1 and OE8) were selected for further study. According to the RT-qPCR data, *PtoHsfB1* expression levels in OE1 and OE8 are 175 and 59 times more than those in WT, respectively.

### Overexpression of *PtoHsfB1* promotes shoot growth

**Figure 3:**
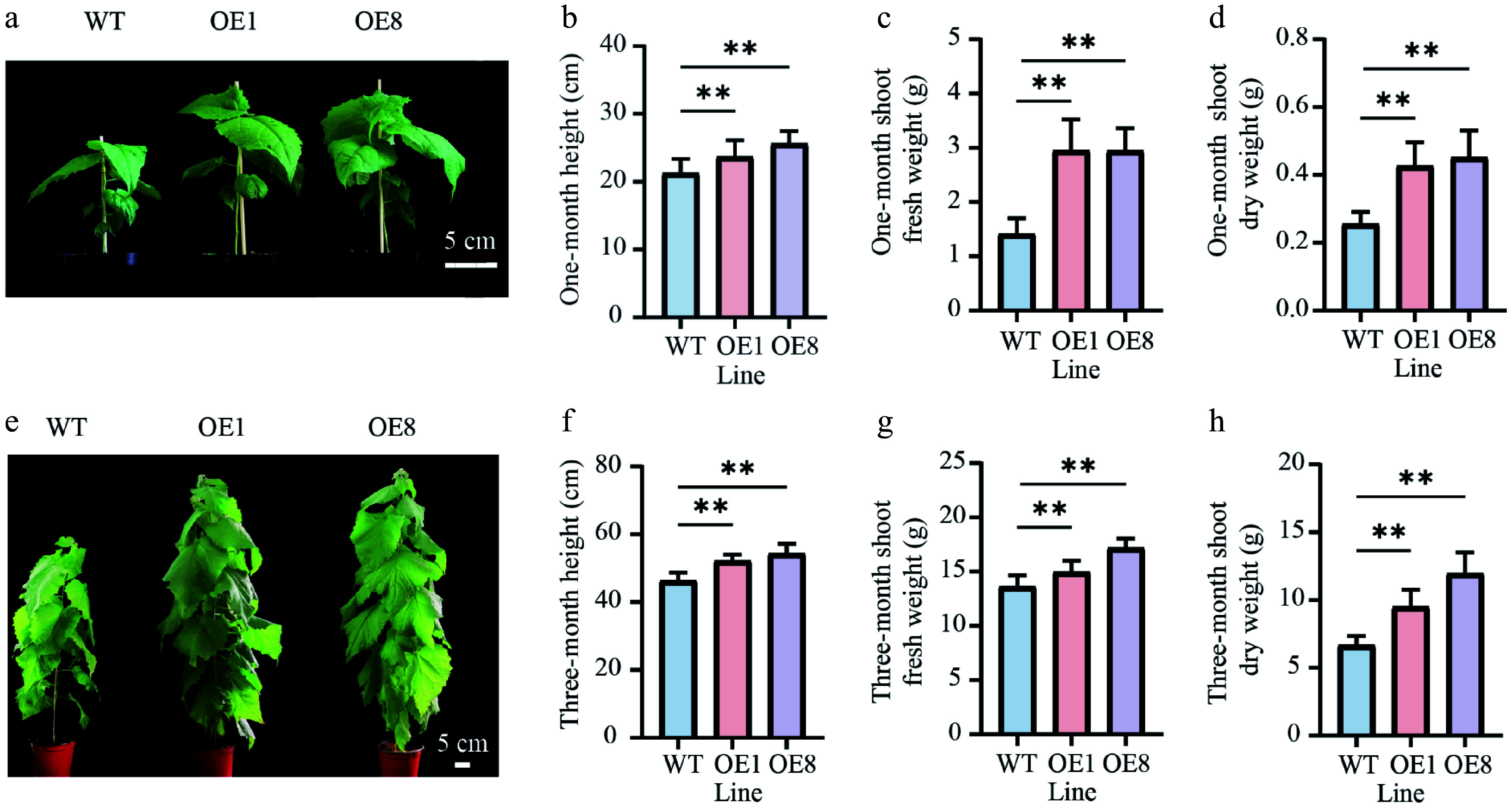
Overexpressing *PtoHsfB1* promoted shoot growth in transgenic poplars. (a) The shoot phenotype of one-month-old plantlets. Scale bar = 5 cm. (b) The height of one-month-old plantlets. (c) The shoot fresh weight of one-month-old plantlets. (d) The shoot dry weight of one-month-old plantlets. (e) The shoot phenotype of three-month-old plantlets. Scale bar = 5 cm. (f) The height of three-month-old plantlets. (g) The shoot fresh weight of three-month-old plantlets. (h) The shoot dry weight of three-month-old plantlets.

To determine whether PtoHsfB1 regulates shoot growth, we compared the shoot phenotype of one-month-old plantlets (Fig. 3a). Phenotypic analysis suggested that the OE lines were significantly taller than the WT (Fig. 3a). The average heights of OE1 and OE8 exceeded those of the WT by 9.17% and 18.35%, respectively (Fig. 3b). Moreover, OE lines produced more leaves and accumulated over twice the shoot biomass of the WT (Fig. 3c and Supplementary Fig. S1). Consistent with the fresh weight measurements, the shoot dry weight was also significantly higher in the OE lines. The shoot dry matter content (dry weight as a percentage of fresh weight) was 81.9% in the WT and at least 84.6% in the OE (Fig. 3c, d). Furthermore, these lines accumulated approximately 1.7 to 1.8 times the shoot dry weight of the WT (Fig. 3c, d). To determine whether the enhanced shoot growth was sustained, we measured the shoot growth metrics of three-month-old plantlets transplanted to the greenhouse using the same methodology as for the one-month-old plantlets. After three months of growth, the growth rate of OE1 and OE8 remained considerably higher than that of WT plants (Fig. 3e). Specifically, the average heights of OE1 and OE8 were 11.3% and 16.2% taller than those of the WT, respectively (Fig. 3f). After three months of growth, OE lines produced more leaves than the WT (Fig. 3e). Their shoot fresh and dry weights were also significantly greater (Fig. 3g, h). The shoot fresh weight in OE1 was 14.97 g, which indicates a 10% increase over the WT, whereas OE8 had a fresh weight of 17.20 g, representing a 26.5% increase relative to the WT (Fig. 3g). Additionally, the shoot dry weights of OE1 and OE8 were 1.5 and 1.8 times greater than that of the WT, respectively (Fig. 3h).

### PtoHsfB1 affects the formation of the cambium and phloem

**Figure 4:**
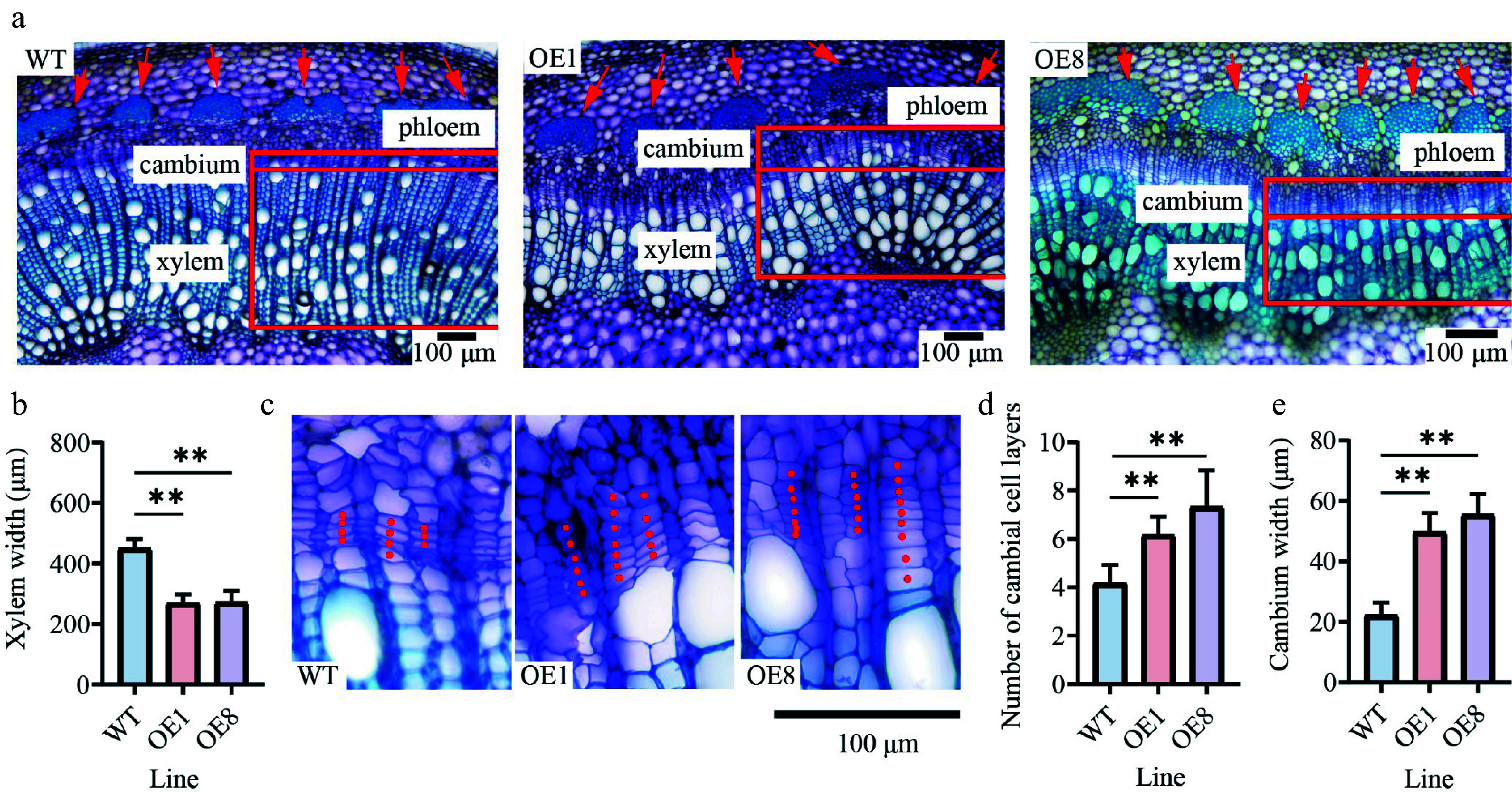
Overexpressing *PtoHsfB1* increases cambial activity and enhances phloem cell growth. (a) Cytological observations of stem cross-sections. The red arrow indicates the phloem. (b) Xylem width. (c) Magnified photos of the cambial area. (d) Number of cambial cell layers. (e) Cambium width.

Since the stems of three-month-old transgenic seedlings were significantly thicker than those of WT plants, we performed tissue section analysis on three-month-old seedlings cultivated in the greenhouse (Supplementary Fig. S2). Cytological analysis of stem cross-sections from the 10^th^ internodes revealed anatomical differences. In OE stems, the phloem cap was more prominent, causing increased cell wall thickness in the phloem fiber cells (Fig. 4a). However, compared with the WT, the xylem widths of the 10^th^ internode in the OE were significantly reduced, indicating that xylem development was suppressed (Fig. 4a). The xylem width of the WT (449.9 μm) was 1.7 times that of OE1, and 1.6 times that of OE8 (Fig. 4b). To determine whether the reduced xylem resulted from changes in cambial activity, a comparison of the cambial zones found that the OE lines had roughly twice as many cambial cell layers as the WT (Fig. 4c, d). The cambium widths of OE1 and OE8 were 2.2 and 2.5 times those of the WT, respectively (Fig. 4e).

### Overexpression of *PtoHsfB1* promotes adventitious root growth

**Figure 5:**
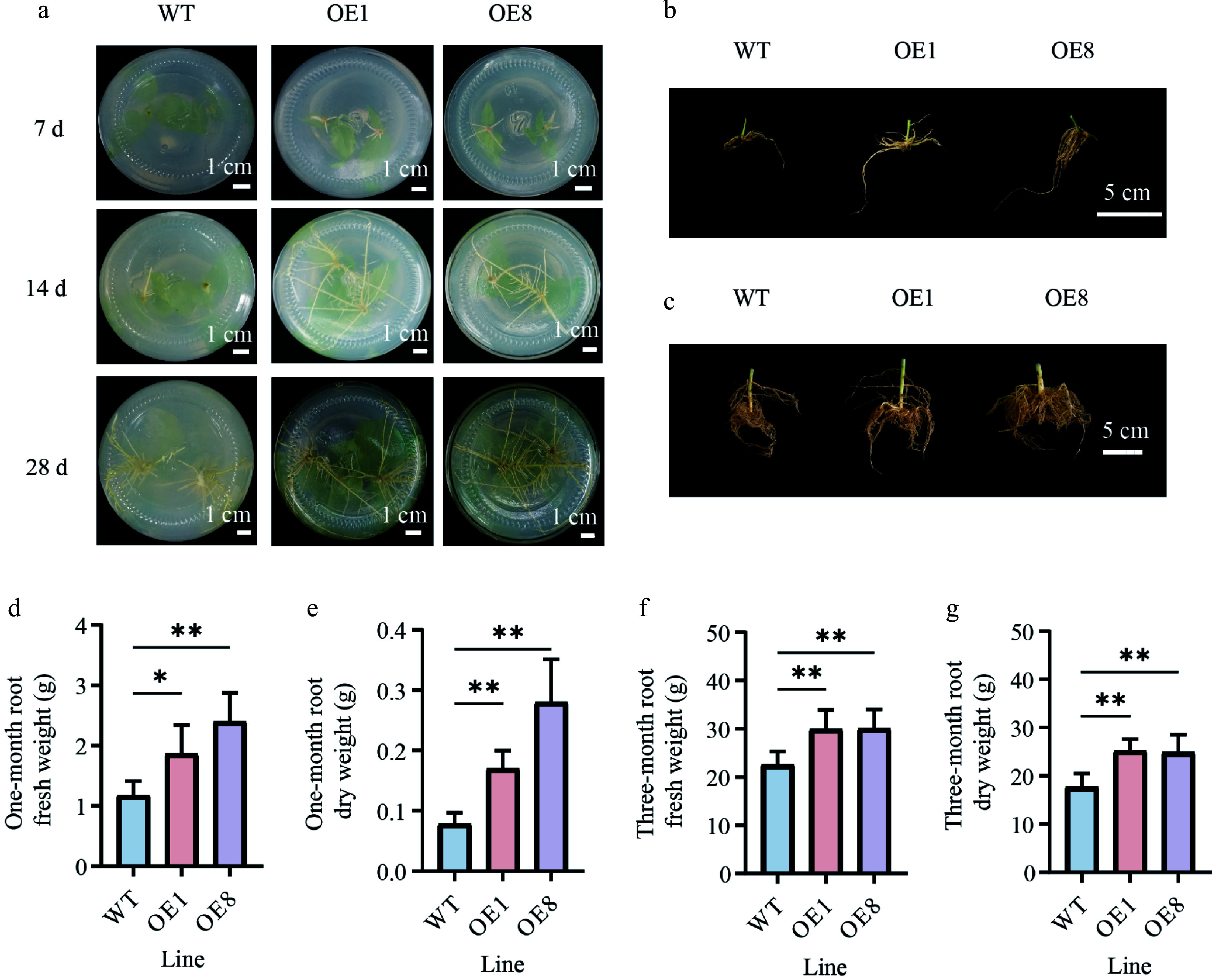
Overexpressing *PtoHsfB1* promoted root growth in transgenic poplars. (a) Growth of adventitious roots in OE and WT plants at different time points under tissue culture conditions. Scale bar = 1 cm. (b) The root phenotype of one-month-old plantlets. Scale bar = 5 cm. (c) The root phenotype of three-month-old plantlets. Scale bar = 5 cm. (d) The root fresh weight of one-month-old plantlets. (e) The root dry weight of one-month-old plantlets. (f) The root fresh weight of three-month-old plantlets. (g) The root dry weight of three-month-old plantlets.

To determine the effect of PtoHsfB1 on root growth, we statistically analyzed root growth indicators and phenotypes. Seven days after inserting branch tips into the rooting medium, adventitious roots had emerged in the transgenic plantlets, but not in the WT. After 14 d, WT plants began to form adventitious roots, while OE plants exhibited a greater number of adventitious roots that were significantly longer. After 21 d, the WT plants had also developed a certain number of adventitious roots; however, both the number and length of the roots were significantly smaller than those in the transgenic lines (Fig. 5a). After 28 d of culture in the rooting medium, we examined the phenotypes of the different lines and found that the OE lines were substantially taller than the WT (Supplementary Fig. S3). Additionally, the root densities of both the one-month-old, and three-month-old transgenic plantlets were much greater than those of the WT (Fig. 5b, c).

In one-month-old plantlets, the OE plants exhibited significantly more roots and higher fresh weight than the WT plants (Fig. 5b, d). The root fresh weights of OE1 and OE8 exceeded those of the WT by 58.97% and 105.13%, respectively (Fig. 5d). The dry matter content of roots was significantly higher in OE plants. The root dry weights of OE1 and OE8 were 2.2 and 3.6 times those of the WT, respectively (Fig. 5e). In the three-month-old plantlets, the root biomass of the OE plants was also significantly greater than that of the WT (Fig. 5f, g). The root fresh weights of the OE plants increased by 27.7% relative to the WT plants (Fig. 5f), and their root dry weights were 1.4 times those of the WT (Fig. 5g). In summary, root biomass (both fresh and dry weight) was significantly higher in the OE than in the WT (Fig. 5).

### Transcriptome analysis of overexpressing *PtoHsfB1*

**Figure 6:**
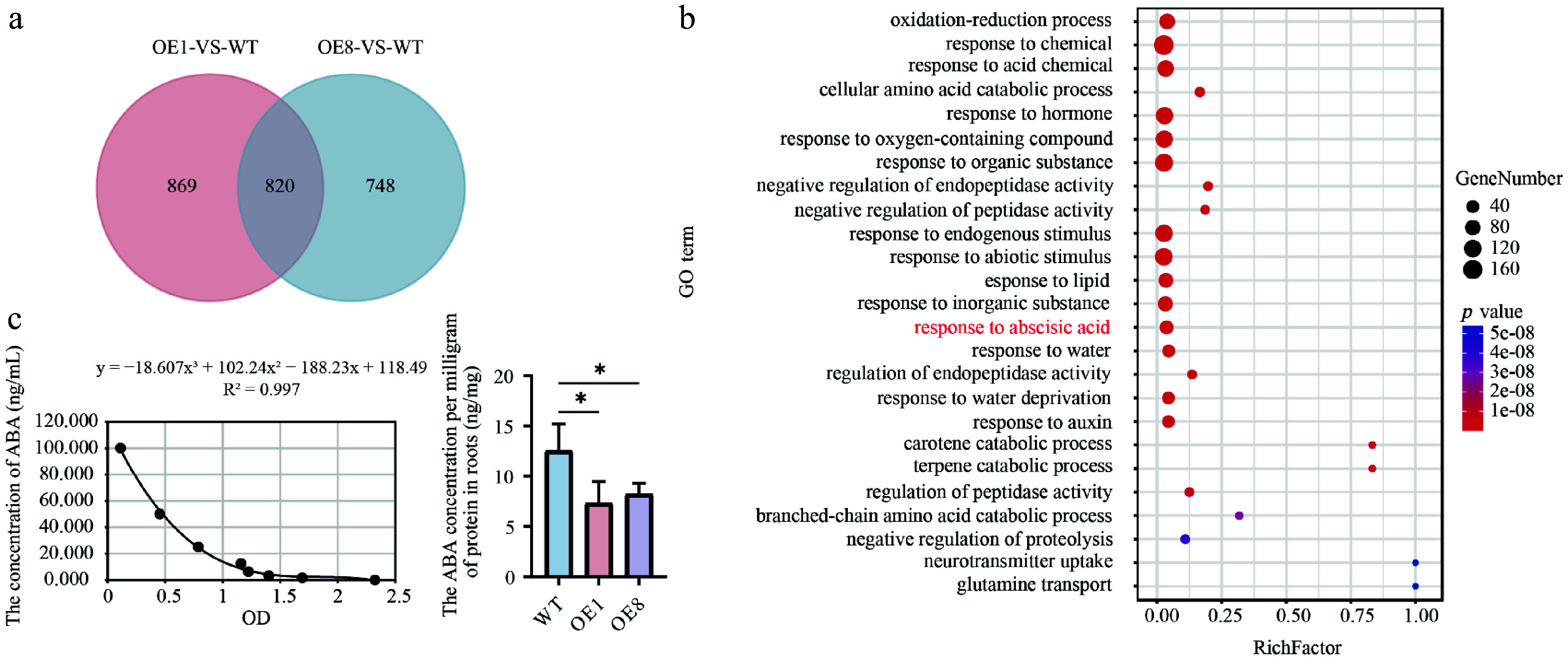
Transcriptome analysis and ABA content detection. (a) Venn diagram analysis of the OE1-VS-WT and OE8-VS-WT comparative groupings. (b) GO enrichment analysis of 820 DEGs depicts dots where their sizes reflect the number of enriched genes. Larger dots indicate a higher gene count. The color of the dots reflects the significance level of the enrichment; dots with a redder hue correspond to smaller Q values, indicating greater significance in the enrichment degree. (c) Left shows the ABA standard curve. The fitting method is polynomial, and the fitting equation is y = −18.607x^3^ + 102.24x^2^ − 188.23x + 118.49, R^2^ = 0.997, right shows ABA content in root tissues.

The root system of plants is crucial for nutrient storage and for providing water and essential nutrients that support shoot growth^[41]^. We performed a comparative transcriptome analysis using the roots of one-month-old WT and OE (*PtoHsfB1-OE1* and *PtoHsfB1-OE8*). Two pairwise comparisons (OE1 vs WT, OE8 vs WT) were performed to study the probable molecular pathways of PtoHsfB1. In the OE1 and OE8, a total of 1,689 and 1,568 significantly differentially expressed genes (DEGs) were identified compared to the WT (Fig. 6a). Comparison of these DEGs identified a total of 820 genes, with 695 down-regulated and 125 up-regulated (Fig. 6a and Supplementary Table S2). An analysis of these 820 DEGs indicated that members of the Aux/IAA family (e.g., POTOM_022766, POTOM_024959, POTOM_057620, POTOM_024235, and POTOM_056487), which act as repressors of early auxin-responsive genes, showed a downward expression trend (Supplementary Table S3).

Gene Ontology (GO) analysis was performed to classify DEGs according to their functions. Three categories were used to group the GO terms: molecular function (MF), cellular component (CC), and biological process (BP). Significant changes in BP were observed in processes linked to development, reproduction, and growth. For CC, major changes occurred in the cell, cell part, and organelle classifications, while for MF, the changes were concentrated in catalytic activity, binding, and nucleic acid binding transcription factor activity (Supplementary Fig. S4). Subsequently, enrichment analysis of GO terms revealed that the top 25 significantly enriched pathways were associated with hormone-related processes, including ABA response (Fig. 6b). Transcriptome analysis suggested that HsfB1 may regulate poplar root growth by modulating the ABA pathway. Subsequently, we measured the ABA content present in root tissues of WT plants and transgenic poplar lines. The findings demonstrated that overexpression of *PtoHsfB1* dramatically reduced ABA content (Fig. 6c).

### PtoHsfB1 is a negative regulator of ABA biosynthesis

**Figure 7:**
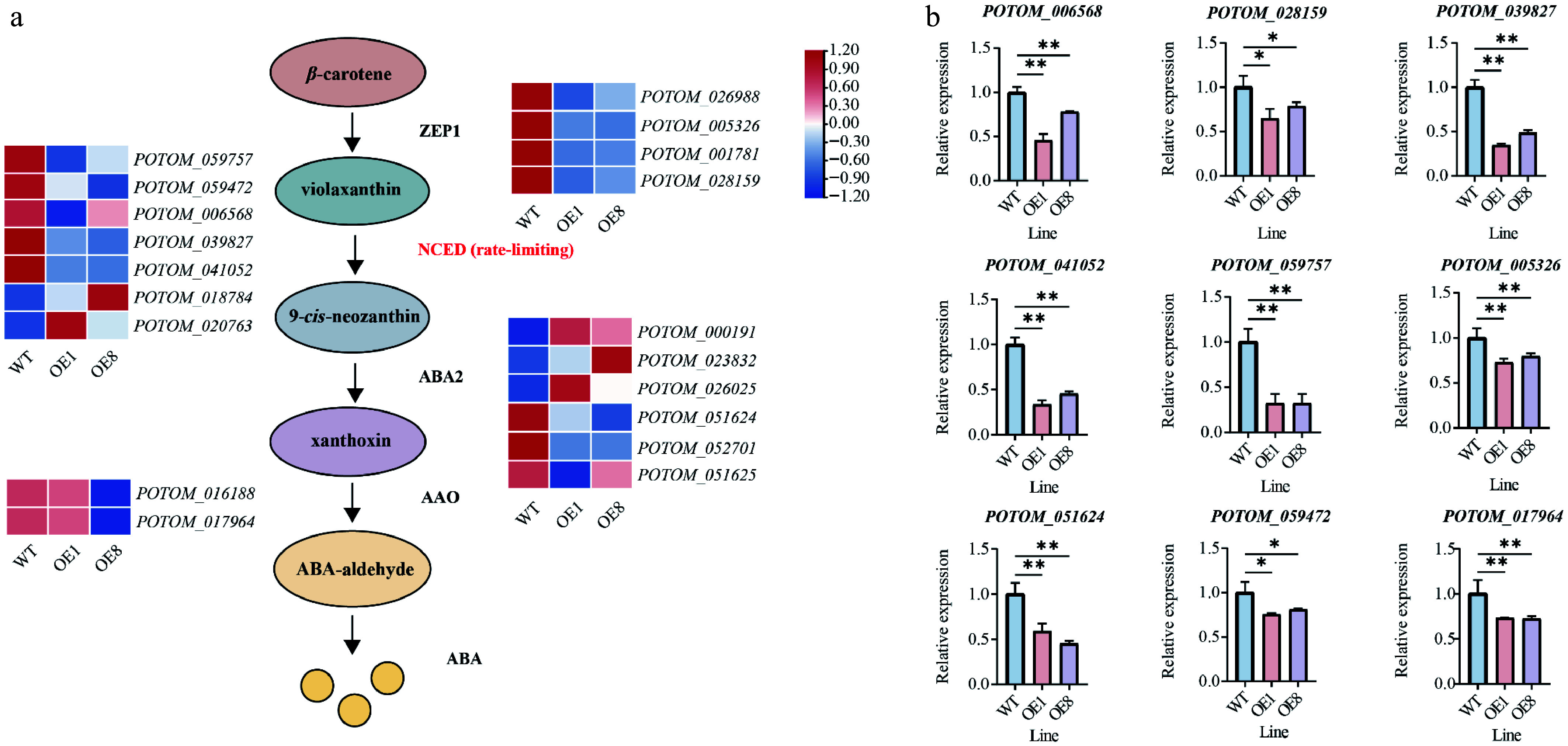
*PtoHsfB1* overexpressing represses ABA biosynthesis. (a) Gene expression is presented in the heatmap. Red and blue represent higher and lower gene expression levels. (b) The relative expression of nine selected ABA biosynthesis related genes.

The regulatory enzymes ZEP1, NCED, ABA2, and AAO are the primary regulators of the ABA synthesis pathway^[17]^. These genes were identified in the *P. tomentosa* genome and their expression changes were analyzed (Fig. 7a). GO enrichment analysis revealed that numerous genes associated with ABA production were significantly downregulated in the transgenic lines (Fig. 7a). A heatmap based on TPM values indicated that members of the ABA synthesis pathway (ZEP1 [4], NCED [5], ABA2 [3], AAO [2]) showed downregulation trends (Fig. 7a). To validate these findings, we randomly selected genes from the ABA synthesis pathway for qRT-PCR analysis (Fig. 7b). Specifically, in the RT-qPCR results, ZEP1 members (*POTOM_005326* and *POTOM_0028159*) exhibited a downregulation trend in the transgenic lines. Similarly, NCED members (*POTOM_039827*, *POTOM_041052*, *POTOM_059757*, and *POTOM_059472*), which encode the rate-limiting enzyme for ABA synthesis, were significantly inhibited in the OE plants. *POTOM_051624* (ABA2) was markedly downregulated in the OE plants as compared to the WT plants. Similarly, the expression of *POTOM_017964* (AAO) was lower in the OE plants (Fig. 7b).

### Overexpressing *PtoHSfB1* leads to salt sensitivity

**Figure 8:**
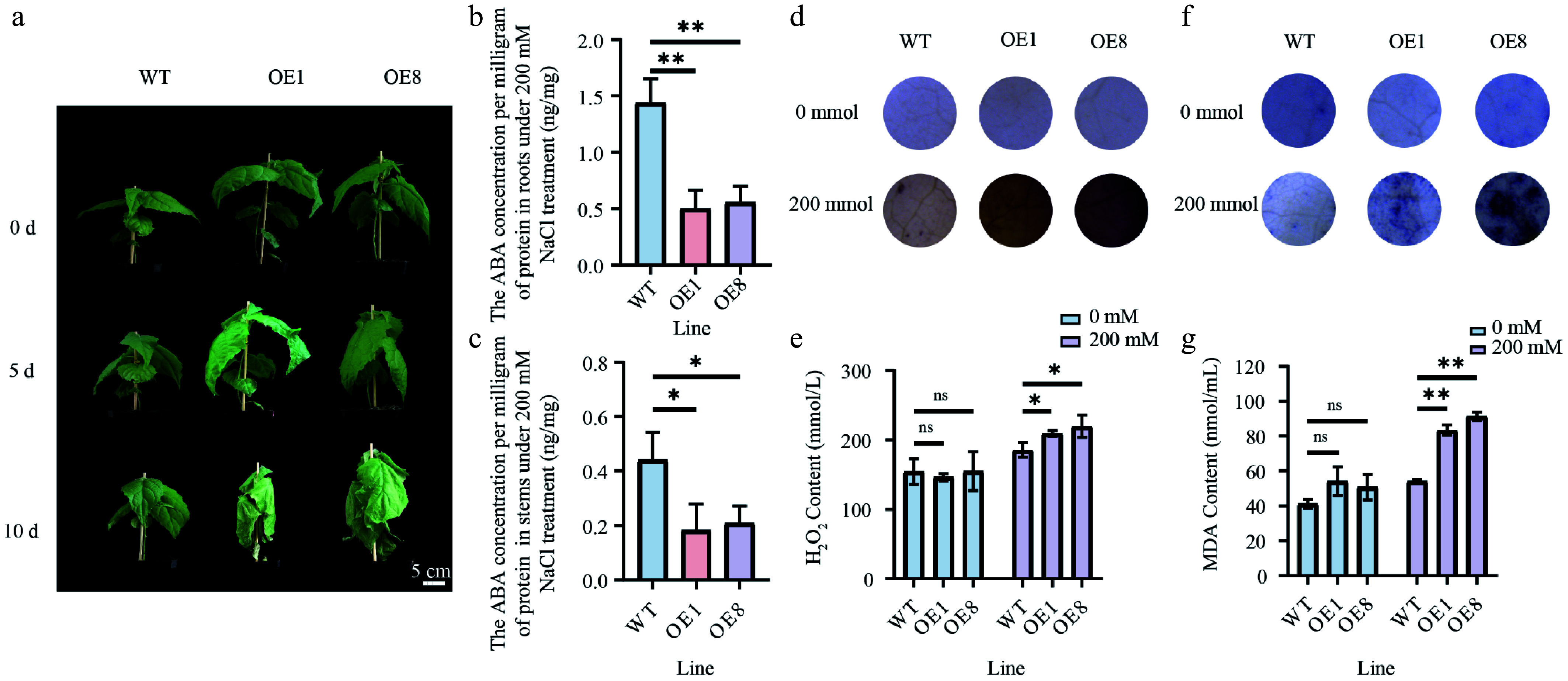
Overexpressing *PtoHsfB1* reduced salt tolerance under 200 mM NaCl. (a) The phenotypes of plantlets. Scale Bar = 5 cm. (b) ABA content in root tissues. (c) ABA contents in stem tissue. (d) DAB staining. (e) H_2_O_2_ content. (f) NBT staining. (g) MDA content.

ABA functions as a critical regulator of plant salt stress responses^[42]^. In this study, the overexpression of *PtoHsfB1* reduced ABA levels (Fig. 6c, Supplementary Fig. S5). Furthermore, transcriptome data from previous studies revealed that PtoHsfB1 is significantly responsive to salt stress^[43]^. To verify this observation, RT-qPCR analyses were conducted on various tissues of *P. tomentosa* subjected to salt stress over different time periods. These results provided preliminary evidence confirming the significant responsiveness of *PtoHsfB1* to salt stress (Supplementary Fig. S6). Therefore, we then examined the salt tolerance of the transgenic poplars. Under non-salt treatment, there were no noticeable phenotypic differences between OE and WT plants. However, after 5 d of treatment with 200 mM NaCl, the number of leaves in the transgenic lines decreased sharply. After 10 d of salt treatment, the leaves of transgenic lines were severely shriveled, and the shoot tips had dried up, whereas the WT leaves showed only slight drooping (Fig. 8a). Furthermore, ABA levels were measured in the root and stem tissues after the treatment with 200 mM NaCl. The findings demonstrated that ABA levels in the OE1 and OE8 lines were significantly lower than in the WT (Fig. 8b, c). We used DAB and NBT histochemical staining to measure changes in H_2_O_2_ and O_2_^−^ concentration in order to gauge the degree of oxidative stress. Under non-salt conditions, DAB staining showed no differences between OE and WT plants. However, after salt treatment, the leaf discs from the transgenic lines exhibited more intense staining than those from the WT (Fig. 8d). Consistent with the DAB staining results, H_2_O_2_ content detection revealed that the OE plants had significantly greater H_2_O_2_ levels than the WT plants following salt treatment, but no significant difference was observed under non-salt circumstances (Fig. 8e). NBT staining revealed that leaf discs from OE plants turned a darker purple than WT plants after salt treatment, indicating higher O_2_^−^ levels in OE plants (Fig. 8f). MDA, a well-known indicator of oxidative stress and lipid peroxidation^[44]^, was also measured to assess the effect of salt stress on cell membrane permeability. Under non-salt treatment, OE and WT plants showed no significant difference in MDA content. However, during salt stress, the MDA levels in OE plants were significantly higher than those in WT plants (Fig. 8g).

## Discussion

Previous studies have demonstrated that Hsf family members play crucial roles in regulating growth and stress responses^[16]^. For example, overexpression of *HsfA5a* in *P. tomentosa* enhances tolerance under salt stress^[5]^. Although the HsfB subfamily is the most highly conserved among the three Hsf subfamilies, its members demonstrate considerable functional diversity^[13]^. In tomato, *HsfB1* functions as both a transcriptional repressor and a co-activator, and its overexpression significantly enhances thermotolerance^[19]^. In *Glycine max*, overexpression of *GmHsfB2b* enhances salt tolerance. In *Populus*, the B subfamily member *PagHsf4* inhibits growth^[21]^. In contrast to previous research, this study demonstrates that overexpression of *PtoHsfB1* in *P. tomentosa* promotes growth but reduces salt stress tolerance. This study expands the understanding of HsfB1 in both plant growth and salt stress response.

Phylogenetic analysis and protein structural characterization indicated that PtoHsfB1 is a typical member of the HsfB subfamily. Its C-terminal region contains the conserved inhibitory core tetrapeptide 'LFGV'^[45,46]^. Our research further confirmed that the core tetrapeptide is essential for the transcriptional repression function of PtoHsfB1. In addition, we demonstrated that overexpression of *PtoHsfB1* promotes growth, as reflected by increased plant height, leaf number, and biomass of both shoots and roots (Fig. 3). The enhanced cambial activity was also observed in *PtoHsfB1*-OE plants. Notably, transgenic lines overexpressing *PtoHsfB1* exhibited a more prominent phloem cap and significantly enhanced thickening of phloem fiber cell walls (Fig. 4). Previous research has shown that the thickening of phloem fiber cell walls enhances the structural integrity of the phloem, facilitates more efficient long-distance transport of photosynthates, and provides protection against herbivores and pathogens^[47]^. Collectively, these findings show that PtoHsfB1 plays a significant role in promoting poplar growth.

In this study, compared with WT plants, *PtoHsfB1* overexpressing plants exhibited significantly more developed root tissues at the tissue culture stage, as well as at the 1-month and 3-month growth stages. Subsequently, transcriptome sequencing was performed on the root systems of 1-month-old greenhouse plants. RNA-seq and corresponding RT-qPCR analyses indicated that the transcriptional levels of ABA synthesis genes were significantly inhibited in *PtoHsfB1*-OE plants. Measurements of ABA content further confirmed that *PtoHsfB1* overexpression reduced endogenous ABA levels. Multiple studies have shown that reduced expression of ABA biosynthesis genes leads to decreased ABA production^[48,49]^, thereby promoting root growth. These research findings are consistent with the results of the present study, collectively supporting the conclusion that reduced ABA content promotes root growth. Therefore, *PtoHsfB1*-OE lines may promote root growth by reducing ABA content.

ABA is not only a vital hormone for growth and development but also a key regulator of abiotic stress responses^[50]^. An increase in ABA content helps enhance plant salt stress resistance, while a decrease in ABA leads to reduced salt stress resistance^[51]^. In the present study, overexpression of *PtoHsfB1* caused a decrease in ABA content. Previous research has firmly established the crucial importance of the Hsf family in enhancing salt tolerance in plants^[52,53]^. However, our findings revealed that HsfB1 functions as a negative regulator of salt stress tolerance rather than a positive one. The analysis of plant wilting and oxidative damage under salt stress further confirmed that *PtoHsfB1*-OE reduces salt tolerance of plants. Moreover, measurement of ABA content before and after salt stress suggested that *PtoHsfB1* overexpression leads to lower ABA levels, which in turn compromised salt stress resistance. Collectively, these findings provide valuable insights into the molecular mechanisms by which PtoHsfB1 regulates salt stress responses through modulation of the ABA pathway.

## Conclusions

This study demonstrates that *PtoHsfB1* functions as a transcriptional repressor in *P. tomentosa*. Overexpression of *PtoHsfB1* significantly promotes the growth of roots, phloem, and cambium. RNA-seq analysis combined with ABA content measurements in *PtoHsfB1*-OE plants indicate that reduced ABA levels may be a key factor driving enhanced poplar growth. Under salt stress conditions, overexpression of *PtoHsfB1* led to decreased ABA accumulation, resulting in reduced salt tolerance, increased oxidative damage, and diminished antioxidant enzyme activity. Collectively, these findings provide a foundation for further investigation into the regulatory roles of the Hsf family in poplar growth, and their underlying mechanisms in salt stress responses.

## SUPPLEMENTARY DATA

Supplementary data to this article can be found online.

## Data Availability

All data generated or analyzed during this study are included in this published article and its supplementary information files.
