# Supplementary material for: PtoHsfB1 regulates growth and salt response by affecting ABA biosynthesis in Populus tomentosa
Source: For Res (Fayettev). 2026 Feb 28;6:e005. doi: 10.48130/forres-0026-0005 (PMC13187910; doi:10.48130/forres-0026-0005)
Supplement: Supplementary file 1 — Supplementary data to this article can be found online. [file forres-6-1-e005-Supplementary.zip › 10.48130_forres-0026-0005-Suppl-FigureS4.pdf]

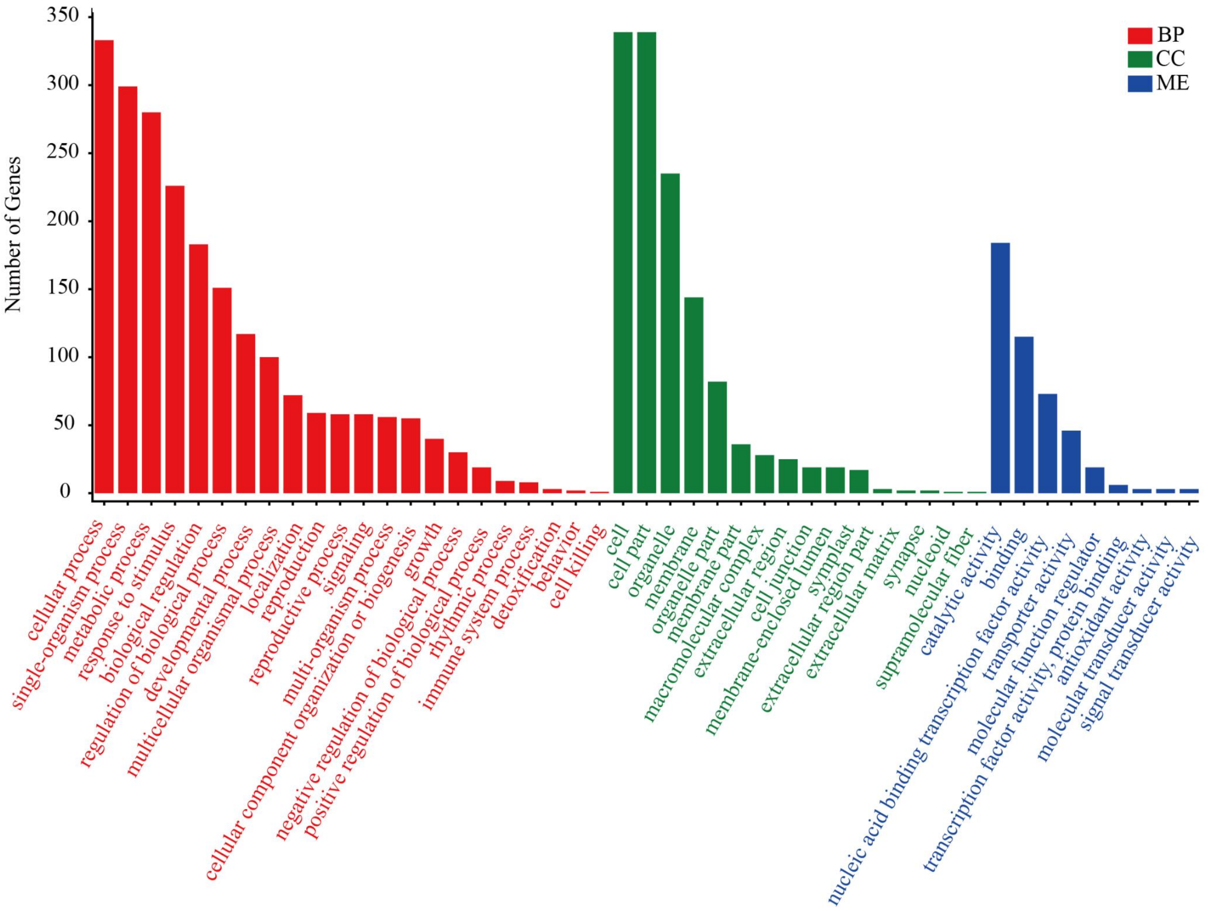

Supplementary Figure S4. GO classifications of DEGs in OE1-VS-WT and OE8-VS-WT. The y- axis indicates the number of DEGs, and the X-axis indicates the top 25 enriched GO terms. The red, green and blue colors represent biological processes, cellular components and molecular function, respectively.
