# Supplementary material for: PtoHsfB1 regulates growth and salt response by affecting ABA biosynthesis in Populus tomentosa
Source: For Res (Fayettev). 2026 Feb 28;6:e005. doi: 10.48130/forres-0026-0005 (PMC13187910; doi:10.48130/forres-0026-0005)
Supplement: Supplementary file 1 — Supplementary data to this article can be found online. [file forres-6-1-e005-Supplementary.zip › 10.48130_forres-0026-0005-Suppl-TableS1.pdf]

Supplementary Table S1. Primers sequences used for gene cloning and for identification of transgenic lines.

Primer names	Primer sequence (5' - 3')
PtoHsfB1-F	5'- GAGAACACGGGGGACTCTAGAATGGCGCAGAGGTCAGCTC -3'
PtoHsfB1-R	5'-GGACTGACCACCCGGGGATCCATTACAGATCTTGATTTCCTTTGCAC-3'
35S	5'-GACGCACAATCCCACTATCC-3'
PtoActin-qF	5'-CTCCATCATGAAATGCGATG-3'
PtoActin-qR	5'-AGCCGTCTCCAGCTCTTGC-3'
PtoHsfB1-qF	5'-GCATCCTCTTCGTCCAACT-3'
PtoHsfB1-qR	5'-TCACCTTGACATACTCCGTC-3'
