# Supplementary material for: PtoHsfB1 regulates growth and salt response by affecting ABA biosynthesis in Populus tomentosa
Source: For Res (Fayettev). 2026 Feb 28;6:e005. doi: 10.48130/forres-0026-0005 (PMC13187910; doi:10.48130/forres-0026-0005)
Supplement: Supplementary file 1 — Supplementary data to this article can be found online. [file forres-6-1-e005-Supplementary.zip › 10.48130_forres-0026-0005-Suppl-TableS2.pdf]

Supplementary Table S2. Primer sequences used for Quantitative real-time PCR verified selected DEG candidates.

Primer names	Primer sequence (5' - 3')
POTOM_059757_qF	5'-CAACAGCCGTGCCTACAAC-3'
POTOM_059757_qR	5'-GGTATCTGGTGGTGGGATCG-3'
POTOM_059472_qF	5'-CTGTCACAACCACACCATCATT-3'
POTOM_059472_qR	5'-GGCTCTACAACTCGTCTATCACT-3'
POTOM_006568_qF	5'-CAACAGCCGTGCCTACAAC-3'
POTOM_006568_qR	5'-TGGGATTAGTAGTAGATGGTGTGTA-3'
POTOM_039827_qF	5'-CAAGCGATCCACTACATACACA-3'
POTOM_039827_qR	5'-AAGCAGTCTCAACAGCATCTAA-3'
POTOM_041052_qF	5'-CCGAAGCAATCCACTACATACA-3'
POTOM_041052_qR	5'-AAGCAGTCTCAACAGCATCTAA-3'
POTOM_005326_qF	5'-GAGGCTCAGGATCTTGATTGC-3'
POTOM_005326_qR	5'-GATGCGGTCACCAGTAACAC-3'
POTOM_028159_qF	5'-AATGGCAAGAATGGCAGCAAT-3'
POTOM_028159_qR	5'-TCGGCAACTCACAGACCTC-3'
POTOM_017964_qF	5'-AGGTGGAGGTGAATCTTCTGA-3'
POTOM_017964_qR	5'-TTCGGTATCGTGTCTATTGTAGG-3'
POTOM_051624_qF	5'-TAAGGCTCCGAGGATATTAGATGTA-3'
POTOM_051624_qR	5'-GGTTGCTGTAATCACACTTCCA-3'
