# Supplementary material for: PtoHsfB1 regulates growth and salt response by affecting ABA biosynthesis in Populus tomentosa
Source: For Res (Fayettev). 2026 Feb 28;6:e005. doi: 10.48130/forres-0026-0005 (PMC13187910; doi:10.48130/forres-0026-0005)
Supplement: Supplementary file 1 — Supplementary data to this article can be found online. [file forres-6-1-e005-Supplementary.zip › 10.48130_forres-0026-0005-Suppl-TableS3.pdf]

Supplementary Table S3. The Aux/IAA genes downregulated in the roots of *P. tomentosa*.

**Downregulated gene IDs**	**WT (TPM)**			**OE1 (TPM)**			**OE8 (TPM)**		
POTOM_056487	7.654506	8.864142	3.836527	1.689638	1.773189	1.446988	1.870907	1.913463	1.314914
POTOM_022766	4.773403	3.770773	1.985674	1.580505	1.402949	1.23171	1.345694	1.151573	0.748686
POTOM_024959	3.33313	2.792397	1.013082	0.902963	0.530663	0.692866	0.791788	0.673698	0.513281
POTOM_057620	5.454212	9.540691	1.857318	2.528296	1.533028	1.44347	1.300795	0.785981	0.798437
POTOM_024235	42.51428	20.00427	4.984004	5.746086	12.74823	6.374621	10.99216	6.198274	8.657687
