# Supplementary figures and images for: PtoHsfB1 regulates growth and salt response by affecting ABA biosynthesis in Populus tomentosa
Source: For Res (Fayettev). 2026 Feb 28;6:e005. doi: 10.48130/forres-0026-0005 (PMC13187910; doi:10.48130/forres-0026-0005)

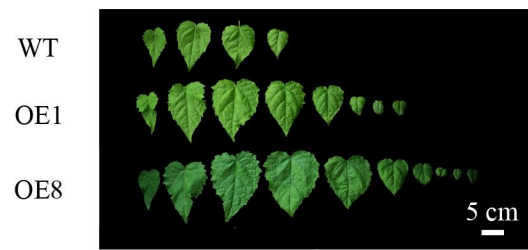

Supplementary Figure S1. Leaf phenotypes and quantities of WT and OE.

Supplement: Supplementary file 1 — Supplementary data to this article can be found online. [file forres-6-1-e005-Supplementary.zip › 10.48130_forres-0026-0005-Suppl-FigureS1.pdf]

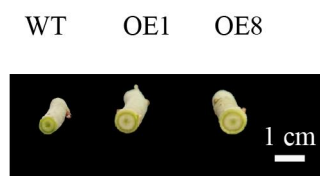

Supplementary Figure S2. Phenotype of stem cross-sections of the tenth internodes.

Supplement: Supplementary file 1 — Supplementary data to this article can be found online. [file forres-6-1-e005-Supplementary.zip › 10.48130_forres-0026-0005-Suppl-FigureS2.pdf]

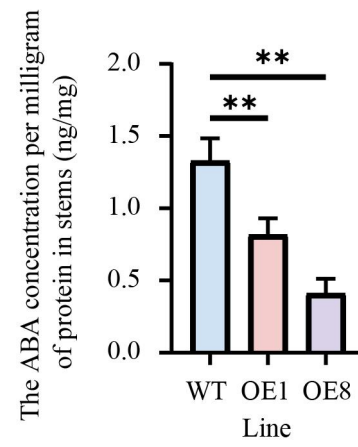

Supplementary Figure S5. ABA content in stem tissues.

Supplement: Supplementary file 1 — Supplementary data to this article can be found online. [file forres-6-1-e005-Supplementary.zip › 10.48130_forres-0026-0005-Suppl-FigureS5.pdf]
